# *Pyricularia graminis‐tritici *is not the correct species name for the wheat blast fungus: response to Ceresini *et al*. (MPP 20:2)

**DOI:** 10.1111/mpp.12778

**Published:** 2019-01-30

**Authors:** Barbara Valent, Mark Farman, Yukio Tosa, Dominik Begerow, Elisabeth Fournier, Pierre Gladieux, M. Tofazzal Islam, Sophien Kamoun, Martin Kemler, Linda M. Kohn, Marc‐Henri Lebrun, Jason E. Stajich, Nicholas J. Talbot, Ryohei Terauchi, Didier Tharreau, Ning Zhang

**Affiliations:** ^1^ Department of Plant Pathology Kansas State University Manhattan KS 66506 US; ^2^ Department of Plant Pathology University of Kentucky Lexington KY 40546 USA; ^3^ Department of Agrobioscience, Graduate School of Agricultural Science Kobe University Kobe 657‐8501 Japan; ^4^ Geobotany, Department of Evolution and Biodiversity of Plants Ruhr‐University Bochum 44801 Bochum Germany; ^5^ UMR BGPI, Université de Montpellier, INRA, CIRAD, Montpellier SupAgro 34398 Montpellier France; ^6^ Department of Biotechnology Bangabandhu Sheikh Mujibur Rahman Agricultural University Gazipur 1706 Bangladesh; ^7^ The Sainsbury Laboratory University of East Anglia, Norwich Research Park Norwich NR4 7UH UK; ^8^ Department of Biology University of Toronto Mississauga ON L5L 1C6 Canada; ^9^ INRA, AgroParisTech UMR BIOGER F78850 Thiverval‐Grignon France; ^10^ Department of Microbiology & Plant Pathology University of California Riverside CA 92521 USA; ^11^ Division of Genomics and Breeding, Iwate Biotechnology Research Center Iwate 024‐0003 Japan; ^12^ Laboratory of Crop Evolution, Graduate School of Agriculture Kyoto University Kyoto 617‐0001 Japan; ^13^ CIRAD, UMR BGPI F‐34398 Montpellier France; ^14^ BGPI, Univ Montpellier, CIRAD, INRA, Montpellier SupAgro F‐34398 Montpellier France; ^15^ Department of Plant Biology Rutgers University New Brunswick NJ 08901 USA

In a review article published in this issue of *Molecular Plant Pathology*, Ceresini *et al. *(2019) wrongly treat the wheat blast fungus as a new species, *Pyricularia graminis‐tritici* (*Pygt*), following the proposal of Castroagudin *et al. *(2016). Despite the host specificity implied by the name *Pygt*, the proposed species concept includes isolates that cause major disease epidemics on finger millet and turf grasses (Castroagudin *et al*., 2017, 2016). These authors also conclude, based on little evidence, that ‘the hypothesis of grass‐specific populations for the overall *Pyricularia oryzae* species complex is falsified’. In addition, they stress that the rice blast fungus, which they describe as *P. oryzae*, ‘may not provide a suitable model for understanding the biology of *Pygt*’. All of these conclusions are misinformed and have serious consequences. International quarantine regulations are needed to block the movement of this fearsome seed‐borne blast fungus through the trade of seed or grain. The *Pygt* designation magnifies the challenge by grouping the dangerous, highly aggressive wheat pathogens from South America and Bangladesh, which are readily distinguishable from other *P. oryzae* lineages, with non‐wheat pathogens that are already found worldwide and are not known to be virulent on wheat or rice. Careful biological analysis of wheat blast host–pathogen interactions has clearly shown that studies of other host‐adapted forms of the fungus are relevant to an understanding of wheat blast and the development of new methods for disease control. Here, we summarize the overwhelming evidence that supports the alternative, internationally recognized designation of *Pyricularia oryzae* (synonym *Magnaporthe oryzae*; Zhang *et al.*, 2016) as a single species divided into host‐adapted lineages with limited primary host ranges. We also delineate the errors that led Ceresini *et al. *(2019) to their false conclusions. The same discussion applies to a second recently published review on the same topic (Ceresini *et al.*, 2018).

Wheat blast disease is caused by a lineage of the ascomycetous fungus *P. oryzae* which is adapted to cause epidemics on hosts in the genus *Triticum* (Cruz and Valent, 2017; Islam *et al.*, 2016; Urashima *et al.*, 1993). This dangerous new disease emerged in Brazil in 1985 and subsequently spread to Bolivia, Paraguay and northern Argentina before moving to Bangladesh in 2016. This disease is poised to spread in South Asia and beyond, with major potential impacts for global food security. *Pyricularia oryzae* is best known as the causal agent of rice blast, which remains a major constraint on the production of rice (*Oryza sativa*) worldwide. In addition, *P. oryzae* causes major diseases on finger millet (*Eleusine coracana*) and foxtail (Italian) millet (*Setaria italica*), which are ancient subsistence crops grown by millions of smallholder farmers in Africa and Asia (Couch *et al.*, 2005, Tanaka *et al*., 2009). Since the 1990s, *P. oryzae* has also caused a damaging turf grass disease on perennial ryegrass (*Lolium perenne*) and tall fescue (*Festuca arundinacea*), first in the USA and now worldwide (Farman *et al.*, 2017; Milazzo *et al.*, 2018; Tosa *et al.*, 2016). Decades of research have shown that distinct *P. oryzae* lineages are responsible for each of these serious recurring blast diseases. These lineages share a very high degree of genome sequence similarity, and have been grouped as a single species (Couch and Kohn, 2002; Gladieux *et al.*, 2018; Klaubauf *et al.*, 2014). The sequence polymorphisms among them have been shown to be relatively minor, although highly consequential. To name them as different species runs counter to accepted taxonomic evidence for species designation, such as reproductive isolation (see below).

**Figure 1:**
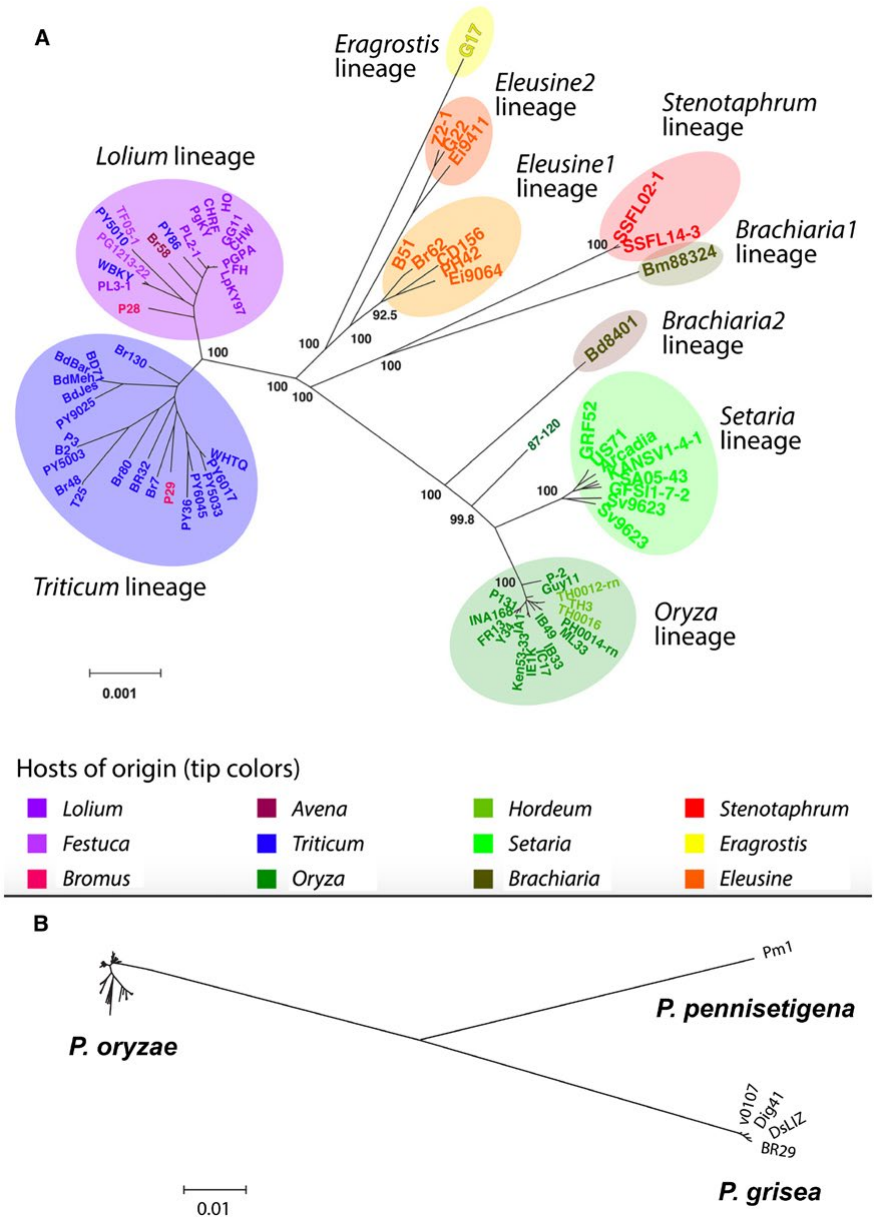
*Pyricularia oryzae* is a single species with major crop‐adapted lineages. (A) Total‐evidence neighbour‐joining distance tree using pairwise distances (number of differences per kilobase of uniquely alignable DNA) calculated from the analysis of pairwise BLAST alignments between repeat‐masked genomes. Only nodes with a confidence of >80% are labelled. Ovals indicate lineages. Branch lengths are proportional to genetic distances between strains. In general, strains isolated from the major crops are associated with host genus‐specific lineages that are conserved worldwide and over time. Shown are 17 *Oryza* strains from eight countries (1948–2009); eight *Setaria* strains from three countries (1975–2005); eight *Eleusine* strains in two known lineages (Tanaka et al., 2009) from six countries (1976–2012); 17 *Lolium/Festuca* strains from three countries (1996–2005); and 20 *Triticum* strains from the four countries with endemic wheat blast (1988–2016). Strain names are colour coded to indicate host of isolation, including three Brazilian *Lolium* strains isolated from wheat or oats, one US *Lolium* strain isolated from wheat, two Paraguayan *Triticum* or *Lolium* strains isolated from *Bromus* spp. in a heavily infected wheat field, and three Thai *Oryza* strains isolated from barley. (B) Neighbour‐joining tree showing the genetic distances separating *P. oryzae* (same relative distances as tree in A), *P. grisea* and *P. pennisetigena*. Distances are in single nucleotide polymorphisms (SNPs) per kilobase. (A) and (B) are reproduced from figs 3 and S4, respectively, from Gladieux et al. (2018). [Colour figure can be viewed at wileyonlinelibrary.com]

A recent in‐depth genomic analysis of a large worldwide collection of *P. oryzae* isolates from different host plants has preserved *P. oryzae* as a single species (Gladieux *et al.*, 2018). This classification is consistent with previous research on host‐adapted forms of the blast fungus, and the last species delineation of *P. oryzae* (Klaubauf *et al.*, 2014). Gladieux *et al.* (2018) analysed the phylogenetic structure within the *P. oryzae* species, relying on whole‐genome sequences from 76 strains collected from 12 host genera, plus five strains from related *Pyricularia* species. Single nucleotide polymorphisms (SNPs) from aligned sequences of 2682 single‐copy, orthologous coding sequences were analysed by discriminant analysis of principal components (DAPC) and maximum likelihood methods. In addition, whole‐genome SNP data were analysed using pairwise distance methods, with special care taken to exclude repetitive sequences, such as diverse transposable elements that show unequal distribution within and between the *P. oryzae* lineages. All analyses by Gladieux *et al. *(2018) were consistent, including the lineage structure from the whole‐genome SNP analysis, reproduced here as Fig. 1A. The maximum pairwise distance between any two *P. oryzae* isolates, including *Oryza* and *Triticum* isolates, was less than 1% genome‐wide. In contrast, consistently greater than 10% differences separated *P. oryzae* and the related species *P. pennisetigena* and *P. grisea*, which cause blast diseases on pearl millet (*Pennisetum glaucum*) and crabgrass (*Digitaria sanguinalis*), respectively (Fig. 1B). Gladieux *et al. *(2018) also reported evidence for the lack of genetic isolation between various host‐specific lineages within *P. oryzae*. Footprints of gene flow between wheat‐infecting isolates and an isolate from another host plant were also reported in another study (Inoue *et al.*, 2017). The extensive study by Gladieux *et al. *(2018) produced compelling evidence that the host‐adapted lineages infecting rice, wheat and other cereal crops are connected by significant and relatively recent genetic exchanges, and therefore correspond to a single species.

The designation of *Pygt* as a separate species by Castroagudin *et al. *(2016) was originally based on erroneous analyses that have been addressed in depth by Gladieux *et al. *(2018)**.** These analyses were based on sequences of 10 housekeeping genes from 79 Brazilian isolates from wheat, 26 isolates from nine other grass species obtained in or near sampled wheat fields, and 23 Brazilian rice isolates (Castroagudin *et al.*, 2016). In this study, 18 of the aggressive wheat isolates from Brazil, plus all but one of the isolates from other grasses, were assigned to *Pygt*. The remaining strains were retained in the *P. oryzae Triticum* and *Oryza* pathotypes. Subsequently, wheat isolates from Bangladesh were included in *Pygt*. The phylogenomic study reported by Gladieux *et al. *(2018) systematically refutes the legitimacy of *Pygt*. First, it re‐examined the 10 genes in the original set of Brazilian strains (Castroagudin *et al.*, 2016) using accepted standards for genealogical concordance for phylogenetic sequence recognition (GCPSR). The GCPSR standard for defining a new species was not satisfied. Only one gene, *MPG1*, supported the *Pygt* grouping, and the other nine genes did not (see fig. S1 in Gladieux *et al.*, 2018). Next, Gladieux *et al. *(2018) examined the 10 gene set using genome sequences from their own global set of strains, which included isolates from the *Lolium* lineage. They obtained the same result: only *MPG1* produced the *Pygt* pattern and not the other genes, and so, again, GCPSR criteria were not satisfied. Moreover, putative *Pygt* strains (as defined by their *MPG1* allele) were discontinuously distributed within all trees built using SNPs in the 2682 orthologous gene set, or with whole‐genome SNP data (see figs 1, 2, 3 and S5 in Gladieux *et al.*, 2018). Within this global strain collection, *Pygt*—as originally defined—includes only a small fraction of the aggressive *Triticum* lineage and mostly comprises isolates from the *Lolium* and *Eleusine* lineages. Clearly, under this scenario, the statement in Ceresini *et al. *(2019) that, ‘*Pygt* is not a wheat‐specialized pathogen’ is logically correct, because *Pygt* includes strains adapted to *Lolium* or *Eleusine* species as their primary hosts. However, the data of Gladieux *et al. *(2018) clearly show that *Pygt*, as defined by the *MPG1* genotype, is an artificial construct because of the use of a single gene genealogy with a specific evolutionary history not reflecting the overall evolution of the species.

In a subsequent analysis, Castroagudin *et al*. (2017) re‐assessed their previous division of aggressive *Triticum* lineage strains into two species, and combined all *Triticum* strains into *Pygt*. This was performed without explicitly stating that the original criteria used to define the species were invalid, and without defining the new criteria used to define their proposed new species. The study in question used SNPs in genome sequences of 95 isolates from wheat, rice and other hosts. In reality, however, the datasets used included only nine isolates from grasses other than wheat or rice that did not group with *Pygt*. These isolates represented three *P. oryzae* lineages and one additional species—*P. grisea*. With so few outgroup members included, these studies are hardly capable of providing an accurate perspective on the relationship between *Pygt* and other host‐specialized forms of *P. oryzae*. In comparison, the study of Gladieux *et al. *(2018) included 26 isolates from 8 different lineages/species.

More importantly, however, the implementation and interpretation of the phylogenetic studies that underpin the new, more inclusive definition of *Pygt* are questionable. The phylogenetic tree in fig. 2 of Ceresini *et al. *(2019) is based on 28 427 genome‐wide SNPs. However, this analysis and those reported previously (Castroagudin *et al*., 2017, Ceresini *et al.*, 2018) use an inappropriate and biased SNP selection procedure that systematically overlooks the nucleotide differences that define the true species boundary between *P. oryzae* and *P. grisea*. Specifically, the first criterion for retaining an SNP in the dataset requires it to be ‘reliably called in the transcriptomic sequences of the Bangladesh sample 12’ (Islam *et al.*, 2016) and ‘genotyped in at least 90% of all other strains’ (Castroagudin *et al*., 2017). By imposing this rule, the authors selected only sites that were polymorphic among *P. oryzae* isolates, but ignored sites that were polymorphic between *P. grisea* and *P. oryzae*, yet monomorphic within *P. oryzae*. This is problematic because there are approximately 10 times as many SNPs between *P. oryzae* and *P. grisea* as there are between any two *P. oryzae* strains. The inevitable result of this SNP selection bias is that *P. grisea* will appear to group within any *P. oryzae* trees built using these datasets. Interestingly, the authors' own data illustrate the problem very well. When they used a multi‐locus genotyping approach, the resulting maximum likelihood trees showed the separate species *P. grisea* and *P. pennisetigena* at the ends of long branches and very well separated from *P. oryzae* (Castroagudin *et al.*, 2016). On the other hand, maximum likelihood trees based on SNP datasets showed *P. grisea* nestling comfortably within the *P. oryzae* trees (Castroagudin *et al*., 2017; Ceresini et al., 2018, 2019). In reality, when using total, unfiltered SNP data, *P. grisea* is highly diverged relative to *P. oryzae*, as illustrated by phylogenomic studies based on a less‐biased, conserved orthologue dataset (see fig. 5 in Gladieux *et al.*, 2018) or, better still for illustrating divergence, whole‐genome pairwise distance data (Fig. 1B).

A second major problem with the definition of *Pygt* is that the species boundary is arbitrary. This is illustrated by the fact that the boundary has changed in all four articles describing the proposed species. If we consider only the trees based on the genome‐wide SNP data, fig. 2 of the review in this journal shows that, with a 28 427 SNP dataset, the isolates from *Rhynchelytrum, Cenchrus* and *Eleusine* group outside of *Pygt*, yet, in Castroagudin *et al*. (2017) (55 041 SNPs) and Ceresini *et al *(2018) (27 961 SNPs), at least one *Cenchrus* and one *Eleusine* isolate group within *Pygt.* In addition, in Ceresini *et al. *(2018), the isolate from *Rhynchelytrum* is placed within *Pygt*. Thus, the species boundary changes depending on how many SNPs are used. Critically, ever since the expansion of *Pygt* to encompass all isolates from wheat, the authors have failed to describe the new criteria that define *Pygt*. In contrast, Gladieux *et al. *(2018) used the Accurate Species TRee ALgorithm (ASTRAL) to test species boundaries by looking for branches that show evidence of genetic isolation. As expected, *P. grisea* occurred on one such branch. The only other branch showing genetic isolation included isolates from *Brachiaria* (*Urochloa*) and *Stenotaphrum* but, with only three isolates being represented, it is premature to define these as belonging to an incipient species.

In response to the data of Gladieux *et al. *(2018), Ceresini *et al. *(2019) argue that GCPSR is too conservative in detecting new species, and may fail to recognize recent speciation events because locus concordance occurs well after reproductive isolation. However, they failed to test for GCSPR, or to discuss the over‐riding factors for dismissing it, and no evidence has been presented for the genetic isolation of *Pygt* strains. In contrast, there is extensive evidence for high sexual fertility (production of abundant perithecia and viable ascospores) among *P. oryzae* lineages and, conversely, for reproductive isolation between *P. oryzae* and *P. grisea* or *P. pennisetigena* (Couch and Kohn, 2002; Kato *et al.*, 2000; Tosa *et al.*, 2006, 2016). ABBA‐BABA tests revealed gene flow across all time scales analysed, and probabilistic chromosome painting showed very clearly that introgressions of chromosome segments from other host‐specialized forms have occurred subsequent to wheat blast emergence (Gladieux *et al.*, 2018). There may be ongoing speciation in this system, and some lineages may end up fusing, hybridizing, or even disappear. Based on current data, however, it is premature to define new species within *P. oryzae*.

Ceresini *et al. *(2019) further suggest that sampling ‘from the same geographical region and in the same time frame’ provides better information on recent speciation than the global collection of strains used by Gladieux *et al. *(2018). This would be true only if the disease had limited geographical distribution. However, the pathogen distribution is global, and global sampling is required to inform international regulations. In addition, sympatric sampling in or near wheat fields has potential problems from opportunistic infections (see below). Gladieux *et al. *(2018) clearly show that, even when comparing supposedly allopatric *P. oryzae* populations, reproductive isolation has not yet occurred for wheat blast. We acknowledge the logical next step would be to combine data from sympatric studies with the global datasets.

The *Pygt* designation significantly complicates quarantine issues. For quarantine restrictions to successfully prevent the entry of a plant pathogen into a new country, it must be possible to precisely name and identify pathogen strains to be excluded. In defining *Pygt*, Ceresini *et al. *(2019) focus mainly on distinguishing wheat pathogens from rice pathogens, ignoring other important, globally distributed *P. oryzae* lineages. The inclusion of *Eleusine* strains in *Pygt* presents an immediate problem for India and Ethiopia, countries at great risk for wheat blast establishment because of favourable environmental conditions, because both countries already have serious problems with *Eleusine* strains on finger millet. Indeed, the general lack of clarity on the status of the *Eleusine* lineages is a major problem because of the importance of this disease. In addition, *Lolium* strains included in *Pygt* already occur worldwide as turf and forage grass pathogens. The *P. oryzae Triticum* lineage stands alone in precisely distinguishing highly aggressive wheat pathogens in South America and Bangladesh from globally distributed *P. oryzae* populations. Careful tracking of movement of *Triticum* strains via accurate DNA‐based diagnostics, seed health protection and international education are needed to block the spread of the deadly wheat blast disease to new production regions. Recently developed polymerase chain reaction (PCR) diagnostic tests (‘MoT3’) distinguish *Triticum* lineage isolates with rare exceptions, which apparently resulted from recombination. This assay is now being used in a number of countries to test potentially infested tissue and grain (Yasuhara‐Bell *et al.*, 2018). To the extent that the MoT3 assay falls short, now or in the future, the correct response will be to develop other, similar tests to distinguish the *Triticum* lineage. Although it is unfortunate that the Linnean binomial for a multi‐host pathogen, such as *P. oryzae*, refers to only a single host, naming a new species is not helpful, or scientifically justified, at this time. *Pyriclaria oryzae* lineages can still be distinguished as such according to the nomenclature of Fig. 1A. Quarantine regulations regularly deal with subpopulation and even strain and clone names.

Ceresini *et al. *(2019) incorrectly assert that the ‘hypothesis of grass‐specific populations for the overall *Pyricularia* species complex is falsified’. This statement requires stronger experimental proof because it goes against decades of research in many laboratories (Couch *et al.*, 2005; Kato *et al.*, 2000; Tosa *et al.*, 2016). The concept of grass‐specific populations refers to the primary host crop on which each adapted lineage is solely responsible for epidemics and losses in farmers' fields. The *P. oryzae* lineages defined by Gladieux *et al. *(2018) support this host specificity, because they precisely align with the historically defined *P. oryzae* pathotypes, which are adapted to a crop and other hosts in the same genus (i.e. finger millet and goosegrass, *E. indica*). The term pathotype was first used by Kato *et al.* (1977) based on the need to differentiate the clearly distinct *P. oryzae* populations devastating finger millet and rice in Japan in the 1970s. The *P. oryzae* pathotype designations are based on decades of pathotyping of *P. oryzae* isolates from different host plants, showing that isolates from rice are the only *P. oryzae* isolates causing epidemics on rice, and they have specific phylogenetic signatures as they all group in a specific phylogenetic clade (Gladieux *et al.*, 2018). Likewise, field isolates collected from *Setaria* spp. and *Eleusine* spp. belong to *Setaria* and *Eleusine* clades, respectively (Gladieux *et al.*, 2018). Isolates collected from *Lolium*/*Festuca* spp. in the USA, Japan, Uruguay and France—including several not in Gladieux *et al. *(2018)—group together in the *Lolium* clade (M. Farman, unpublished data; Milazzo *et al.*, 2018). Thus, phylogenomic studies do not support the existence of widespread cross‐infections among these hosts in the field.

Ceresini *et al. *(2019) do not present compelling evidence to support their strong statements on the lack of host specificity in *P. oryzae*. Their assertions appear to be based, in large part, on the isolation of wheat pathogenic strains from 13 different grass genera in or near infected wheat fields. However, with the exception of wheat, barley, signalgrass (*Brachiaria*), oats and rice, they failed to perform laboratory inoculations to rule out opportunistic infection by *Triticum* strains on secondary hosts in spore‐rich environments in or near heavily infected fields under disease‐conducive conditions. In laboratory studies, adapted fungal strains rapidly kill primary host plants because nearly all penetration sites result in large coalescing lesions. In contrast, the fungus causes fewer, often smaller, lesions on secondary, non‐adapted hosts because only a few penetration sites succeed and colonization is reduced at successful sites (Cruz and Valent, 2017). Indeed, Castroagudin *et al*. (2017) reported that their grass isolates were highly aggressive on wheat and showed the same virulence groups as the wheat isolates. This suggests that they are *Triticum* isolates undergoing opportunistic infections, similar to two *Bromus* strains isolated in an infected wheat field in Paraguay (Fig. 1A). The existence of host‐specific populations cannot be falsified without obtaining a suitable number of additional isolates from all putative hosts and performing quantitative, confirmatory inoculations on all hosts.

Ceresini *et al. *(2019) even dispute that wheat is the primary host for wheat isolates. They speculate that, ‘If non‐wheat hosts such as *Urochloa* (*Brachiaria*) are shown to be the primary host for the pathogen, while the blast disease on wheat is mainly collateral damage that occurs due to inoculum spillover from an epidemic on the non‐wheat host under particular environmental conditions, it may be more appropriate to breed for resistance in the non‐wheat host’. However, their own laboratory inoculations (Castroagudin *et al.*, 2016) show that these strains kill wheat plants in the same assays in which they cause isolated lesions on *Brachiaria* and other hosts, refuting the collateral damage argument and strategies arising from it. Indeed, greatly enhanced aggressiveness of recently collected *Triticum* strains compared with strains collected soon after the fungus jumped to wheat in Brazil in the 1980s (Inoue *et al.*, 2017) demonstrates the fine‐tuning or adaptation of *Triticum* strains to wheat (Cruz *et al.*, 2016). Although some *Lolium* strains can infect wheat, they are less aggressive than early *Triticum* strains (Farman *et al.*, 2017). It is, however, critical to understand all potential secondary hosts for *Triticum* strains, because alternative hosts can contribute inoculum for the wheat crop and serve as reservoirs for the long‐term survival of the pathogen and for recombination between strains of different pathotypes (Kato *et al.*, 2000; Langner *et al*., 2018).

Ceresini *et al. *(2019) repeatedly assert that, ‘*P. oryzae* may not provide a suitable model for understanding the biology of *Pygt*’. We agree that each *P. oryzae* lineage responsible for serious crop epidemics must be evaluated independently for the management of disease. However, strains of all *P. oryzae* lineages form pressurized appressoria for host penetration and use the same invasion strategy (Zellerhoff *et al*., 2006). The analyses of complete genomes of *Oryza*, *Setaria*, *Triticum* and *Eleusine* isolates showed that more than 88% of the genes involved in the interaction with the plant, including putative effectors, have homologues in all genomes (Chiapello *et al.*, 2015; Yoshida *et al.*, 2016). Comparison of reference genome sequences for *Triticum* and *Oryza* strains show seven conserved core chromosomes plus supernumerary minichromosomes (Peng *et al.*, 2018). Strains in different lineages, including the *Oryza* and *Triticum* lineages, are sexually fertile, producing abundant viable ascospores in laboratory studies (Tosa *et al.*, 2006, 2016). Sufficient similarities exist to indicate that the first question for the study of any aspect of wheat blast disease should reasonably be: ‘Is it like rice blast?’ Many current research strategies to breed or genetically engineer resistance to wheat blast are based on knowledge transfer from the well‐studied rice pathosystem.

In conclusion, based on the strong evidence, *Pygt* cannot be considered as a distinct species. Because all strains within *Pygt* form a subset of the strains in *P. oryzae*, we formally treat *Pygt* as a synonym of *P. oryzae* according to the code of nomenclature of plants, fungi and algae (see below). Resulting from this formal synonymization, *P. oryzae*, and not *Pygt*, is the valid name for the wheat blast fungus. Nevertheless, it is critically important to closely monitor further evolution in *P. oryzae*. Real danger lies in the potential for *Triticum* strains now in South Asia to recombine with sexually fertile *Oryza* strains in the Himalayan foothills (Saleh *et al.*, 2014) to produce strains that are aggressive pathogens of both rice and wheat. Although there are currently no *P. oryzae* lineages adapted to barley or oats, these could evolve if epidemics become common in these crops (Urashima *et al*., 2017). The potential for the evolution of new threats from *P. oryzae* should not be underestimated.

***Pyricularia oryzae*** Cavara, Fungi Longobardiae Exsiccati 1: no. 49, 1891. – Type: on *Oryza sativa*, Italy, Trovamala.

= *Pyricularia graminis‐tritici *Castroag., S.I. Moreira, Maciel, B.A. McDonald, Crous & Ceresini, Persoonia 37: 211, 2016. – Holotype: Brazil, Goiás, isolated from head of *Triticum aestivum*, 2012, J.L.N. Maciel, HISA 10298 (syn. nov.).
